# Antibodies with specificity to glycan motifs that decorate OMV cargo proteins

**DOI:** 10.1128/msphere.00907-24

**Published:** 2025-02-26

**Authors:** Hyun Young Kim, Christina M. Rothenberger, Mary E. Davey, Manda Yu

**Affiliations:** 1 Department of Microbiology, ADA Forsyth Institute, Cambridge, Massachusetts, USA; 2 Department of Oral Microbiology and Immunology, School of Dentistry, Dental Research Institute, Seoul National University, Seoul, South Korea; 3 Department of Oral Microbiology, University of Florida College of Dentistry, University of Florida, Gainesville, Florida, USA; University of Nebraska Medical Center College of Medicine, Omaha, Nebraska, USA

**Keywords:** *P. gingivalis*, OMV, IgM antibodies, O-glycosylation, PG1881

## Abstract

*Porphyromonas gingivalis* is a major etiological agent of periodontal disease, and infections with this bacterium are associated with systemic pathologies, including atherosclerosis, rheumatoid arthritis, and Alzheimer’s disease. *P. gingivalis* has a variety of immune evasion mechanisms and exhibits highly variable cell surface characteristics that are strain dependent, complicating the development of effective vaccines and therapeutics. Here, we show that a subset of immunoglobulin M (IgM) antibodies in antiserum raised against *P. gingivalis* strain W83 selectively recognize the outer membrane vesicles (OMVs). Pre-adsorption with a mutant strain lacking an OMV-specific lipoprotein (PG1881) that has been shown to be glycosylated significantly enhanced IgM specificity toward PG1881 and the OMVs. In addition, the IgM reactivity against the OMVs derived from a mutant lacking enzymes required for O-glycosylation was markedly reduced, indicating that the IgM targets the glycan motifs on proteins carried on OMVs. Importantly, the IgM exhibited specific recognition of OMVs from both *P. gingivalis* and *Porphyromonas endodontalis*, while showing low reactivity toward other genera belonging to the phylum Bacteroidetes. This study revealed a potential host evasion strategy and highlights the potential for utilizing O-glycans in vaccine development and OMV-targeted antibodies in therapeutic interventions to combat *P. gingivalis* infections.

**IMPORTANCE:**

O-glycosylation of cell surface proteins by bacteria is known to play a role in various functions including colonization and immune evasion. This study highlights the identification of IgM antibodies that specifically recognize O-glycosylated proteins that are selectively carried on outer membrane vesicles (OMVs). The findings suggest a potential host evasion mechanism and open new avenues for using OMVs in vaccine development and targeting O-glycans with antibodies as a therapeutic strategy against the subgingival pathobiont *P. gingivalis*.

## INTRODUCTION

Bacterial extracellular vesicles (EVs) play a crucial role in cell-to-cell communication among bacteria and in the manipulation of host immune responses (1). In gram-negative bacteria, the term “outer membrane vesicle” (OMV) is commonly used to describe EVs that are generated from the outer membrane. The oral bacterium *Porphyromonas gingivalis* is prolific in the production of OMVs, and these EVs have been shown to impact a wide variety of basic biological activities and the host immune response (2–7). Studies have also provided compelling evidence linking *P. gingivalis* OMVs to several systemic diseases. A prominent area of research is the association between *P. gingivalis* OMVs and atherosclerosis, due to their potent platelet aggregation activity (8) and the induction of low-density lipoprotein aggregation (9). Additionally, OMVs from *P. gingivalis* have been associated with increased risks of Alzheimer’s disease (8), rheumatoid arthritis (10), and nonalcoholic fatty liver disease (11). As *P. gingivalis* is considered a major etiological agent of chronic periodontitis (12), these findings are particularly concerning because patients with periodontitis may face an additional risk factor for developing these systemic diseases.

A variety of immunogens on the *P. gingivalis* cell surface are being considered for vaccine development. This includes specific outer membrane proteins (13), the K-antigen capsule (14), the gingipains (15), and the fimbriae (16). Furthermore, the OMVs themselves are being considered in large part because they are structurally more stable than proteins demonstrated by proteinase K resistance and the ability to withstand long-term storage (16–18). Lastly, the lipopolysaccharides (LPS) along with a number of the anionic polysaccharide-modified proteins present on the OMVs are also regarded as potential vaccine targets (19).

Recently, a fimbriae-like lipoprotein (PG1881) was identified that is selectively enriched on OMVs and is required for the formation of *P. gingivalis* aggregates (biofilms) on red blood cells (20). In addition, this fimbriae-like protein has been shown to form fibrillar structures and be heat-labile (21). Importantly, PG1881 is highly conserved between *P. gingivalis* strains as well as across *Porphyromonas* species (21, 22) and, as mentioned, is intentionally packaged into OMVs. Our studies have provided a clue that PG1881 plays a role in OMV integrity. Specifically, while the PG1881 mutant is still able to produce OMVs, when the OMVs were examined by TEM, the OMVs were oversized and irregular in shape, suggesting that the OMVs lack membrane integrity (20). Importantly, and likely linked to its folding, stability, and function, this OMV-specific lipoprotein undergoes various posttranslational modifications, including O-glycosylation, which involves the addition of sugars to serine (S) or threonine (T) residues (20, 23). Recent work determined that the biosynthesis of the O-glycan requires two glycosyltransferases, PGN_1134 and PGN_1135 (also known as PG1345 and PG1346 in strain W83), which add the two terminal N-acetylhexosamine (HexNAc) sugars (23, 24). Notably, our studies demonstrated that the export of PG1881 to OMVs is impaired when the protein is not appropriately O-glycosylated—specifically, when PG1345 and PG1346 are absent (20). Altogether, the data indicate that glycosylation is highly relevant to PG1881 folding, stability, and function. While the functional role of O-glycosylation remains unclear, a few other surface-exposed proteins, including Type 9 Secretion System (T9SS) proteins, lipoproteins, and outer membrane β-barrel proteins, are also O-glycosylated (23). Also, related to PG1881 localization is synthesis of sphingolipids (SLs) by *P. gingivalis*. Specifically, the SL-null mutant ΔPG1780 produces OMVs that lack PG1881 (7).

For a previous study, we generated antiserum in rabbits immunized with formalin-fixed whole cells of W83, an encapsulated strain of *P. gingivalis* (25), and a high titer of IgM to K-antigen capsule was detected (25–28). Importantly, B cells generate different classes of antibodies, with IgM serving as the first line of defense against pathogen invasions by binding and activating the complement system (29), but unlike protein antigens, polysaccharides often elicit an IgM response without undergoing isotype switching to IgG. In addition, capsules can mask the cell surface and thereby limit an IgG response to cell surface proteins making encapsulated pathogens unrecognized by immune surveillance. Intriguingly, the respiratory pathogen *Moraxella catarrhalis* can redirect immune responses by triggering IgM production away from the cell by deploying its superantigen-bearing OMVs (30), highlighting how bacteria can manipulate the immune system via the interactions between released extracellular OMVs and IgM antibodies, a mechanism that limits contact between the bacteria and the host.

Pre-adsorption is an effective technique for removing unwanted antibodies that may interfere with analytical outcomes (31) or target specificity (32). Here, we employed pre-adsorption methods to eliminate non-OMV-binding IgM (including K-antigen IgM) using ΔPG1881 mutant cells. The pre-adsorbed IgM antibodies showed strong cross-reactivity with OMVs, yet significantly reduced reactivity to the OMVs produced by the glycosylation-defective mutant (ΔPG1345-PG1346), indicating that the OMV-targeting IgM specifically recognizes O-glycosylation on the OMVs produced by *Porphyromonas* species. This finding provides further evidence that *P. gingivalis* can manipulate the human immune system via the release of its OMVs and also provides a foundation for the development of a detection method for *P. gingivalis* OMVs in various tissues, aiding in the investigation of the relationship between *P. gingivalis* and systemic diseases. Ultimately, the variable regions of the IgM could be identified for monoclonal antibody production, while the specific O-glycans may serve as a target for vaccine development, paving the way for various therapeutic and diagnostic applications.

## RESULTS

### IgM antibodies detect OMVs from both W83 and ATCC 33277

**Fig 1:**
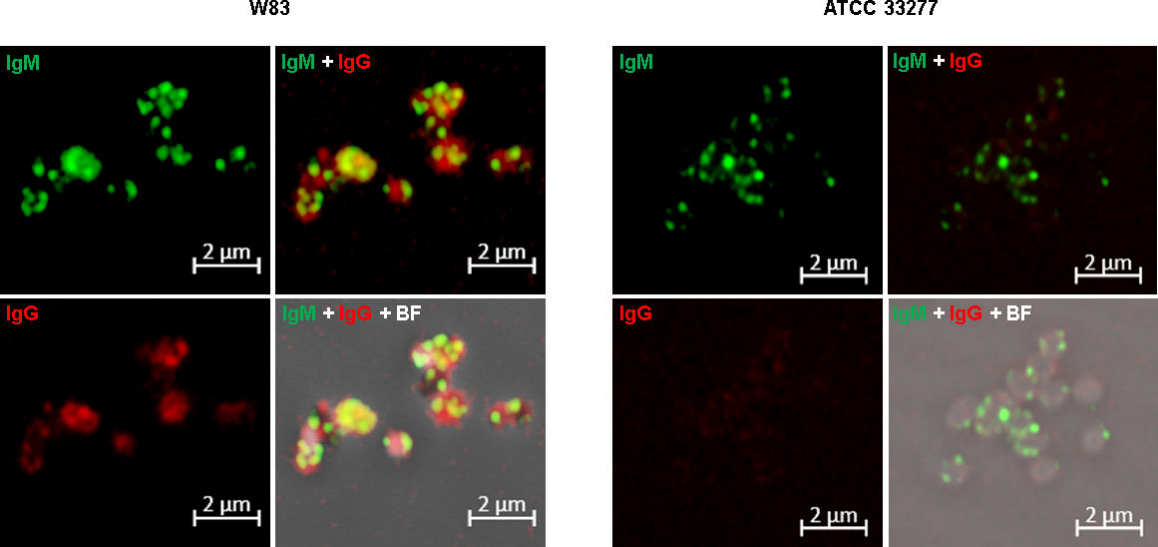
Immunofluorescence microscopy of *P. gingivalis* strain W83 and ATCC 33277 using anti-W83 antiserum. IgG antibodies in the antiserum were labeled by Alexa Fluor 647 (red), and secondary Alexa Fluor 488-labeled anti-IgM antibodies (green) were used to detect the IgM antibodies in the antiserum. IgM antibodies in the antiserum target OMV-like structures on both W83 and ATCC 33277 strains (BF, brightfield)

Anti-W83 whole-cell antiserum was treated with the Alexa Fluor kit for labeling the IgG antibodies with AF647 (red fluorescence), while AF488-conjugated anti-IgM secondary antibodies were used to detect the IgM that bound to the *P. gingivalis* surface (green fluorescence). The encapsulated strain W83 and nonencapsulated strain ATCC 33277 were used for comparison of antibody binding. The results (Fig. 1) show that both IgM and IgG have stronger signals on the surface of the W83 cells than on the ATCC 33277 cells. This aligns with the expectation since the antiserum was raised to W83 cells. Surprisingly, ATCC 33277 demonstrated only low levels of IgG binding, suggesting that there are surface features of W83 cells, such as outer membrane proteins, lipids, or polysaccharides that are missing or presented at lower levels on the cell surface of strain ATCC 33277. We observed that some IgM signals overlapped with the IgG signals on the W83 cells (yellow color in IgM+IgG panels), yet not to the ATCC 33277 cells, reflecting the presence of IgM targeting K-antigen capsule only on W83 cell surface (25–27). Importantly, spherical structures with strong IgM signals were observed on the cells of both the W83 and ATCC 33277 strains (green color in the IgM+IgG panels), indicating that a subset of IgM antibodies in the antiserum was preferentially interacting with antigens on the OMVs, regardless of the strain, suggesting that the antigens present on the OMVs are conserved across different strains.

### Pre-adsorbed IgM antibodies enhanced the specificity for OMV binding

**Fig 2:**
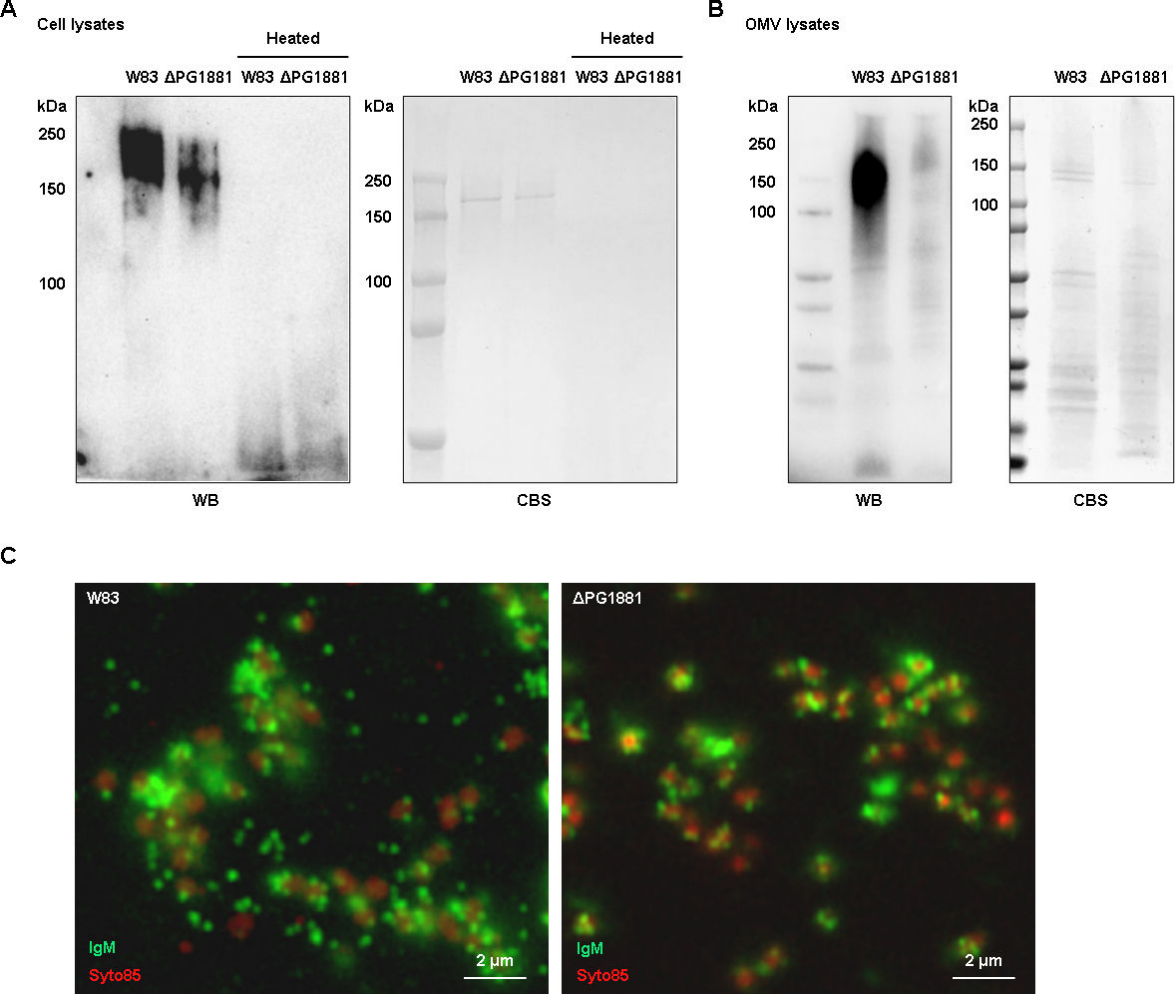
Pre-adsorbed IgM antibodies recognize OMVs of *P. gingivalis*. Western blot analysis of cell lysate (**A**) or OMV lysate (**B**) of W83 and the ΔPG1881 mutant using the pre-adsorbed antiserum as the primary antibodies and anti-IgM-HRP antibodies as the secondary antibodies to detect binding of IgM. (**C**) Immunofluorescence microscopy images of W83 and the corresponding ΔPG1881 mutant cells treated with the pre-adsorbed antiserum and then with anti-IgM-AF488 (green). The cells were also stained by the fluorescent nucleic acid stain SYTO85 (red) (WB, Western blot; CBS, Coomassie blue staining)

**Fig 3:**
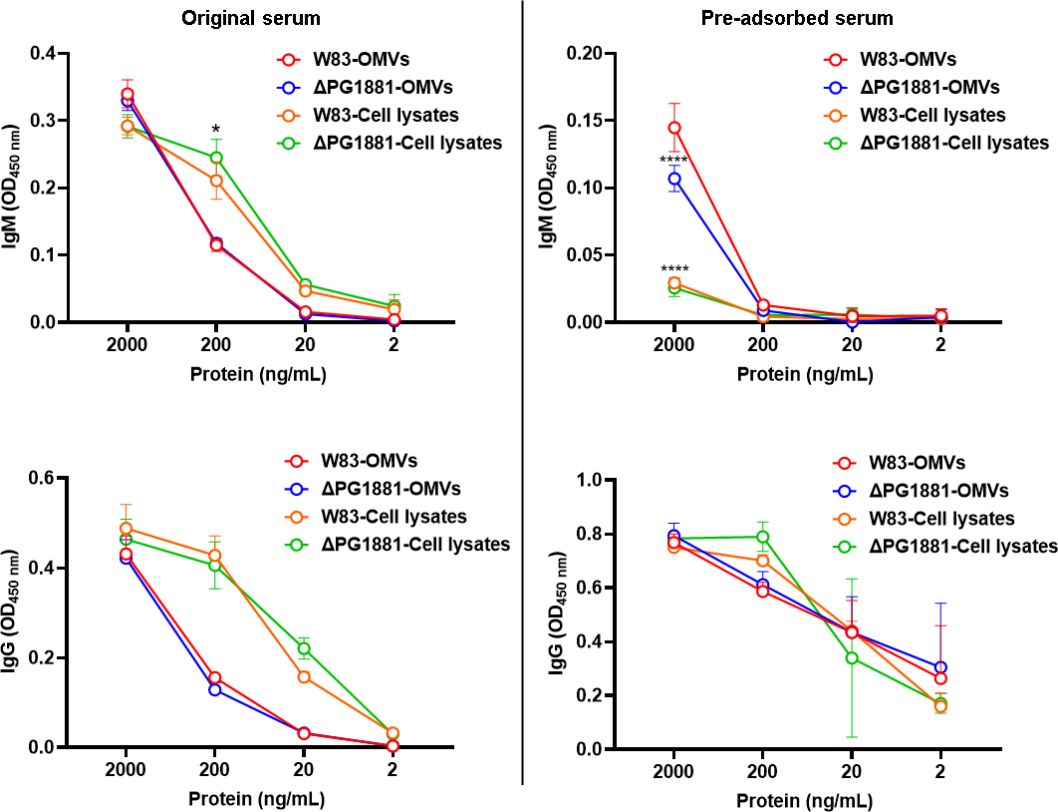
Pre-adsorbed IgM shows a significant reduction in binding to W83 cell lysates. ELISA using original antiserum (left panel) or pre-adsorbed antiserum (right panel) as the primary antibodies followed by either IgM-HRP or IgG-HRP as the secondary antibodies to detect the antigens on crude OMVs and cell lysates. The graphs are shown as the mean values ± standard deviations. Statistical significance was determined by two-way ANOVA with Dunnett’s multiple comparison test. *P <0.05 compared to W83 cell lysates and ΔPG1881 cell lysates (left panel). ****P < 0.001 compared to W83-OMVs or W83 cell lysates (right panel).

When considering the identity of the conserved antigens on the OMVs of *P. gingivalis*, we speculated that the OMV-enriched lipoprotein PG1881 could be a candidate (20). To purify PG1881-specific IgM and deplete K-antigen-targeted IgM antibodies, the antiserum was pre-adsorbed with the W83 ΔPG1881 mutant cells. To determine if this pre-adsorbed IgM targeted the PG1881 protein, we examined the *E. coli* recombinant PG1881 protein that was shown to be recognizable by the anti-PG1881 antibodies in our previous study (20). However, no signal was detected at the molecular size of monomeric PG1881 (around 50 kDa) (Fig. S1), indicating that the purified IgM does not bind to the PG1881 protein directly. Since IgM antibodies typically recognize lipid or polysaccharide antigens, we reasoned that the antigen was likely a modification on this lipoprotein that was only generated when the protein was produced by *P. gingivalis*. Indeed, when cell lysates were examined, a strong signal was detected, yet at a higher molecular weight than the monomeric size of PG1881 and only when there was no heat applied to the lysate (Fig. 2A). This suggests that PG1881 may form a homo-oligomer or hetero-oligomer. Furthermore, the signal from the W83 ΔPG1881 cell lysates was much weaker than that of the parent strain W83, indicating that indeed PG1881 is recognized by the IgM antibodies, but there are likely additional lipoproteins on the OMVs with the same modification. To directly test the signal on the OMVs, an OMV lysate was analyzed, here a strong signal was observed with the range of 100 kDa–250 kDa in the W83 OMVs, but it was absent in the W83 ΔPG1881 OMVs (Fig. 2B). Furthermore, when we repeated the immunofluorescence microscopy using the pre-adsorbed antiserum with W83 and ΔPG1881 (Fig. 2C), we observed OMV fluorescence signals in the extracellular space between the WT cells but absence in the ΔPG1881 mutant and the negative control (Fig. S2). To further verify the western blot and the immunofluorescence microscopy data, we compared the reactivity of the original antiserum and pre-adsorbed antiserum by enzyme-linked immunosorbent assay (ELISA) together with IgM-HRP or IgG-HRP as the secondary antibodies (Fig. 3). The pre-adsorbed antiserum showed a significant reduction of IgM reactivity to the cell lysates of W83 and ΔPG1881 mutant, indicating the pre-adsorption had effectively removed most of the cell surface binding IgM antibodies. In contrast, the IgM reactivity remained strong to the OMVs with a slightly higher signal to the WT OMVs than to the ΔPG1881 mutant OMVs. On the other hand, we did not see the difference between the original and pre-adsorbed antiserum in IgG reactivity against OMVs and cell lysates, suggesting that the IgM but not the IgG antibodies in the antiserum can be segregated by the pre-adsorption method for enhancing the specificity of OMV binding.

### O-linked glycosylation is the likely target of the IgM antibodies

To determine the nature of the OMV antigens recognized by the IgM antibodies, we employed a variety of strains with defects in surface component and OMV cargo proteins, such as strains with T9SS-related genes, including peptidylarginine deiminase (PPAD) and mfa5 (Fig. S3) and the K-antigen null mutant ΔPG0106 (Fig. S4), and found that there was no significant difference between the mutants and the WT OMVs. Since earlier studies determined a strong link between the synthesis of sphingolipids (SLs) and localization of PG1881 on OMVs, strains altered in the synthesis of SLs were also examined. Studies have determined that SLs are a key feature of surface lipids of *P. gingivalis* (6) that affect the presentation of surface polysaccharides (33) as well as the protein profiles of OMVs (7). We therefore compared the different sphingolipid biosynthesis mutants ΔPG1348 and ΔPG1780, and both the OMVs and TCA-extracted proteins from the OMVs of these mutants showed no difference in reactivity of IgM with WT (Fig. S5). The above results support our working model that the IgM antibodies recognize a modification on OMV cargo proteins, not the proteins themselves nor the SLs, which are also carried on the OMVs.

**Fig 4:**
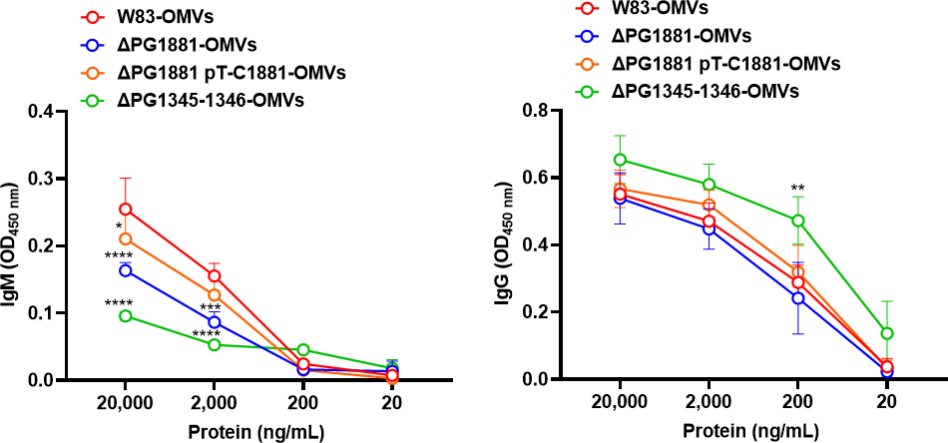
Pre-adsorbed IgM showed a significant reduction in binding to purified OMVs from the O-glycosylation-deficient mutant. ELISA using the antiserum that was pre-adsorbed with W83ΔPG1881 mutant cells as the primary antibodies, followed by either IgM-HRP or IgG-HRP as the secondary antibodies to detect the antigens on purified OMVs from W83, ΔPG1881, PG1881 complementary strain (ΔPG1881 pT-C1881), and the O-glycosylation-deficient mutant (ΔPG1345-1346). Representative results from three biological replicates are shown. Representative results from three biological replicates are shown. The graphs are shown as the mean values ± standard deviations. Statistical significance was determined by two-way ANOVA with Dunnett’s multiple comparison test. *P < 0.05, **P < 0.01, ***P <0.001, ****P < 0.001 compared to the W83-OMVs.

Studies have shown that a number of OMV cargo proteins are O-glycosylated and that the glycosyltransferases encoded by PG1345 and PG1346 are required (23, 24); therefore, the reactivity of IgM to the OMVs of the mutants ΔPG1345-PG1346, Δ PG1881, and the PG1881 complementary strain were examined. In other parts of this study, crude OMVs were used and were sufficient to provide conclusive results; however, to exclude the possibility of residual protein aggregates in the crude OMVs, we opted for density gradient-purified OMVs for these experiments. The result shows that IgM reactivity is significantly reduced in the ΔPG1345-PG1346 mutant OMVs (Fig. 4, left panel). The reactivity to the ΔPG1881 OMVs was reduced and could be recovered in the complemented strain. In contrast, the IgG reactivity was similar in the tested strains except for the higher signal in the ΔPG1345-PG1346 mutant OMVs (Fig. 4, right panel). This result indicates that the IgG target is not related to O-glycosylation, yet the defect in glycosylation may expose more IgG antigens.

### The IgM antibodies show specificity to *Porphyromonas* species

**Fig 5:**
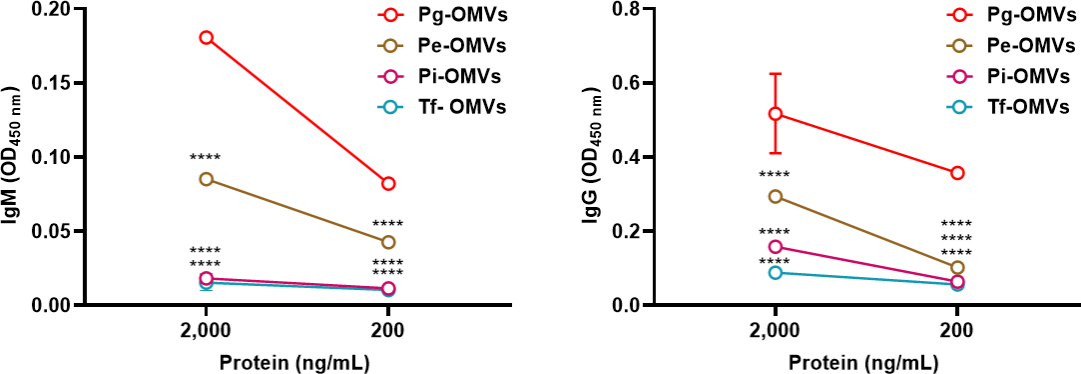
Pre-adsorbed IgM shows specific binding to crude OMVs from *P. gingivalis* and *P. endodontalis*. ELISA using PG1881 mutant cells pre-adsorbed antiserum as the primary antibodies and followed by either IgM-HRP or IgG-HRP as the secondary antibodies to detect the antigens on the crude OMVs from oral Bacteroidetes. The graphs are shown as the mean values ± standard deviations. Statistical significance was determined by two-way ANOVA with Dunnett’s multiple comparison test. ****P < 0.001 compared to the Pg-OMVs group at the same concentration.

To determine whether the IgM antibodies are specific to the OMVs from *P. gingivalis*, a variety of other members of the phylum Bacteroidetes were examined, including species belonging to *Porphyromonas*, *Prevotella*, and *Tannerella*, which are key periodontal health-related bacteria (34, 35). OMVs derived from *Porphyromonas endodontalis* ATCC 35406, *Prevotella intermedia* strain 17, *Tannerella forsythia* ATCC 40437, and *Porphyromonas gingivalis* W83 were examined by ELISA. The results (Fig. 5) show that there was a strong IgM binding to the *P. gingivalis* W83 OMVs, while binding to *P. endodontalis* OMVs was reduced at the concentration of 2000 ng/mL. The binding of IgM to *P. intermedia* OMVs and *T. forsythia* OMVs was much weaker at 2000 ng/mL and closed to background levels at 200 ng/mL. The IgG antibodies show a similar trend as IgM at 2000 ng/mL, but the signal for *P. endodontalis* was similar to the signals of *P. intermedia* and *T. forsythia* at 200 ng/mL. The results indicate that the IgM antibodies in the pre-adsorbed antiserum have a higher preference for the OMVs from *Porphyromonas* species than the two other members of the Bacteroidetes, demonstrating a potential for specific detection of OMVs from *Porphyromonas* species in a complex specimen.

## DISCUSSION

Glycans on the surface of bacterial cells influence an array of interspecies and host-microbe interactions, and studies have shown that they are fundamental to oral microbiome structure and function (36). The impact is evident in variations in cell-associated capsular polysaccharides, S-layers, LPS molecules, and glycosylated lipoproteins, as well as exopolysaccharides (EPS) that are shed into the surroundings. In general, these cell surface glycans impact colonization and biofilm development. EPS and OMVs are critical components of the matrix formed by surface-associated microbial communities (37), and most relevant to this study is an abundance of OMVs, and polymeric material encasing the cells has been reported for subgingival plaque matrix (38). Since the extracellular matrix is a defining attribute of biofilms that plays a fundamental role in housing and protecting the microbiota, determining the types of glycans and the interactions between matrix glycans and the immune system is critical.

The primary finding of this study is the discovery of IgM antibodies and that *P. gingivalis* releases OMVs into its surroundings that carry glycan-modified (O-glycosylated) lipoproteins that are primarily recognized by IgM. Although IgM is the predominant antibody during a primary challenge to an antigen, unlike IgG, an IgM response is typically not long-lasting (39). Importantly, polysaccharides are known to stimulate the production of IgM without eliciting an isotype switch to IgG. In general, the presentation of surface polysaccharides can go unrecognized and potentially cloak the bacterial cell, avoiding recognition and immune activation. Since the O-glycosylation mutant (∆PG1345-1346) used in this study demonstrated stronger IgG binding than the parent strain W83, we propose that O-glycosylation may represent a mechanism that masks proteins and prevents immune recognition. In addition, by releasing OMVs with glycosylated cargo proteins, *P. gingivalis* may be similar to *Moraxella catarrhalis*; it is able to redirect an immune attack away from the bacterial cell by triggering IgM production to its OMVs (30).

Due to various immune evasion strategies employed by *P. gingivalis*, this species can persist in the subgingival region of the oral cavity, avoiding elimination by the immune system. This prolonged presence of *P. gingivalis* poses a significant threat to health, particularly in relation to periodontitis, but as noted also in relation to systemic health. Timely detection of *P. gingivalis* can offer valuable insights into an individual’s potential health risks. However, the genetic and phenotypic diversity that exists among *P. gingivalis* strains leads to significant variability in the cell surface properties and thereby potentially detection (40). One key surface structure that is variable is K-antigen capsule, e.g., strains W83 and W50 strains are encapsulated, whereas ATCC 33277 and 381 strains lack K-antigen capsule (41). Hence, IgG antibodies raised from different strains may exhibit strain-specific reactivity. In contrast, the highly conserved O-glycosylation antigens across the different strains may be ideal for *P. gingivalis* detection. We propose that the discovery of the IgM characterized in this study provides further support for using anti-glycan antibodies in research, diagnostic, or potential therapeutic applications to combat *P. gingivalis* infections. It has been shown that IgM generated by pneumococcal polysaccharide vaccine is sufficient to confer protective immunity to *Streptococcus pneumoniae* (42); therefore, the use of O-glycan antigens on the cell surface or *P. gingivalis* OMVs for vaccine development for the prevention of *P. gingivalis*-related systemic diseases such as Alzheimer’s disease is worth further investigation. These antibodies could be used to investigate the role of *P. gingivalis* OMVs in the pathogenesis of systemic diseases and as a therapeutic in controlling periodontitis and other related chronic inflammatory diseases.

## MATERIALS AND METHODS

### Bacterial strains and culture conditions

*P. gingivalis* strain W83 and ATCC 33277 were grown on trypticase soy agar plates supplemented with 5 µg/mL hemin, 1 µg/mL menadione, and 5% defibrinated sheep blood (Northeast Laboratory Services) (BAPHK) at 37°C in an anaerobic chamber (Coy Lab Products) with an atmosphere containing 5% hydrogen, 10% carbon dioxide, and 85% nitrogen. Planktonic cultures of *P. gingivalis* were grown in trypticase soy broth (Becton, Dickinson and Company) supplemented with 5 µg/mL hemin and 1 µg/mL menadione (TSBHK).

### Immunofluorescent staining and confocal microscopy

*P. gingivalis* cells were grown on BAPHK plates and were harvested and resuspended in PBS. The cell density was adjusted to OD_600_ = 1.8, and ethanol was added to make the final concentration to 70% ethanol and incubated for 30 min. After incubation, 1 mL of the cell suspension was pelleted down by centrifugation at 6,500 × *g* for 1.5 min. The pellet was resuspended in 1 mL of PBS-T (Tween 20 at 0.1%) with 1% BSA and incubated for 30 min with shaking. The cells were pelleted and resuspended in 500 µL of PBS-T with 1% BSA and Alexa Fluor 647-labeled (Invitrogen) W83 antiserum (1:100) and incubated for 30 min. The cells were washed twice with PBS-T and resuspended in 500 µL of anti-rabbit IgM-AF488 antibody (Abcam) (1:500) and incubated for 30 min. The cells were washed twice in PBS-T and resuspended in 30 µL for laser confocal microscopy analysis. For sample with unlabeled pre-adsorbed antiserum, 1 µL SYTO85 (Invitrogen) was added during secondary antibody incubation. The Alexa Fluor-labeled kit is not suitable for IgM labeling (information from manual mp20180 of Invitrogen) and optimized for IgG labeling. The AF-647 signal reflects the binding of IgG, while AF488 signal reflects the binding of IgM to the targets. The labeled sample was observed with Zeiss LSM 880 confocal upright microscope equipped with a 32-channel GaAsP Airyscan detector for super-resolution capabilities. Transmitted light images were captured using the T-PMT detector.

### Pre-adsorption of antiserum with *P. gingivalis* ΔPG1881cells

Overnight culture of *P. gingivalis* W83 ΔPG1881 cells was harvested by centrifugation and washed with PBS. The cell suspension was adjusted to 70% ethanol and incubated for 30 min with shaking. The cells were then resuspended to OD_600_ = 3 in PBS. In the pre-adsorption mixture, 200 µL of ethanol-treated cells and 200 µL of anti-W83 antiserum were mixed in 0.8 mL of PBS with protease inhibitors (Halt protease inhibitor, Thermo Fisher Scientific) and incubated at 4°C for 30 min. The cells were removed by centrifugation at 12,000 × *g* for 3 min. Fresh 200 µL of ethanol-treated cells was added to the supernatant and repeated the binding step. The pre-adsorbed serum was mixed with 1 mL of glycerol (50% final concentration) and stored at −20°C.

### Immunoblots

Crude OMVs and whole cell lysate as well as recombinant PG1881 were prepared as described previously (20). For western blot analysis, proteins were separated and transferred onto a PVDF membrane. The membrane was blocked in PBS-T with 1× Pierce Clear Milk Blocking Buffer (Thermo Fisher Scientific). Primary anti-W83 or PG1881 antiserum was added at 1:1000 dilution and incubated with the membrane for 1 h with rocking at room temperature. The membrane was washed and incubated with either HRP-conjugated anti-rabbit IgG or IgM (Abcam) at 1:3,000 dilution for 1 h with rocking at room temperature. The membrane was washed in PBS-T before detection using SuperSignal West Pico Chemiluminescent Substrate (Thermo Fisher Scientific). A representative image from at least two individual experiments is presented.

### Outer membrane vesicles

For OMV purification, all bacterial strains used in this study were cultured at 37°C in an anaerobic chamber for 16 h in trypticase soy broth supplemented with 5  µg/mL hemin and 1  µg/mL menadione (TSBHK). After culturing, the optical density (OD_600_) was 1.0–1.2 for *P. gingivalis* (W83, ATCC 33277, and 381), 1.0–1.2 for *P. endodontalis* ATCC 35406, 1.0–1.2 for *P. intermedia* strain 17, and 0.5–0.7 for *T. forsythia* ATCC 43037. Bacterial OMVs were purified as described previously with minor modifications (43, 44). Bacterial culture supernatants were collected by centrifugation (10,000 × *g*, 4°C, 30 min) using a F12-6 × 500 rotor (Thermo Fisher Scientific Inc., Waltham, MA, USA) and concentrated (4,000 × *g*, 4°C, 30 min) using a 100 kDa cutoff centrifugal filter (Merck Millipore, Darmstadt, Germany). The filtrates were washed with PBS and subjected to ultracentrifugation (120,000 × *g*, 4°C, 2 h) using a Type 45 Ti rotor (Beckman Coulter, Brea, CA, USA) to isolate crude OMVs. To obtain purified OMVs, the crude OMV pellet was resuspended in 40% OptiPrep (0.9 mL; Sigma, St. Louis, MO, USA), overlaid with 35% OptiPrep (1.55 mL) and 10% OptiPrep (1.55 mL), and subjected to buoyant density gradient ultracentrifugation (160,000 × *g*, 4°C, 4 h) using an SW 60 Ti rotor (Beckman Coulter). Density fractions (400 µL each) were obtained from the top of the gradient (#1–10), and fraction #5 was washed with PBS (3.6 mL) by ultracentrifugation (120,000 × *g*, 4°C, 2 h) using an SW 60 Ti rotor (Beckman Coulter). The purified OMVs were dissolved in 200 µL of PBS and stored at −80°C until use. Protein concentration of the OMVs was quantified using the bicinchoninic acid assay (Thermo Fisher Scientific Inc.).

### Enzyme-linked immunosorbent assay

A direct ELISA using anti-*P*. *gingivalis* W83 serum was conducted as described previously (11). OMVs and bacterial cell lysates were coated onto a high binding 96-well plate (Greiner Bio-One, Kremsmünster, Austria) at 4°C for 16 h. The antigen-coated plate was washed four times with TBST (Tris-buffered saline containing 0.1% Tween 20) and blocked with 1% BSA in PBS at room temperature for 1 h. After four additional washes with TBST, the plate was incubated with anti-*P*. *gingivalis* serum (1:200 dilution) at room temperature for 2 h, followed by four washes with TBST. To detect antigen-binding IgG and antigen-binding IgM, HRP-conjugated goat anti-rabbit IgG (1:20,000 dilution) and HRP-conjugated goat anti-rabbit IgM (Abcam; 1:5,000 dilution) were applied, respectively. After incubation at room temperature for 20 min, the plate was washed four times with TBST. Signal development was performed using 1-Step TMB ELISA substrate solutions (Thermo Fisher Scientific Inc.), and the reaction was stopped with 2 N H_2_SO_4_. The absorbance was measured at 450 nm using a SpectraMax (Molecular Devices, San Jose, CA, USA).

## SUPPLEMENTAL MATERIAL

Supplemental figures msphere.00907-24-s0001.pdf Figures S1 to S5.

ASM does not own the copyrights to Supplemental Material that may be linked to, or accessed through, an article. The authors have granted ASM a non-exclusive, world-wide license to publish the Supplemental Material files. Please contact the corresponding author directly for reuse.
