# Supplementary material for: Antibodies with specificity to glycan motifs that decorate OMV cargo proteins
Source: mSphere. 2025 Feb 26;10(3):e00907-24. doi: 10.1128/msphere.00907-24 (PMC11934327; doi:10.1128/msphere.00907-24)
Supplement: Supplemental figures — Figures S1 to S5. [file msphere.00907-24-s0001.pdf]

Supplementary Data:

Supplementary Figure S1

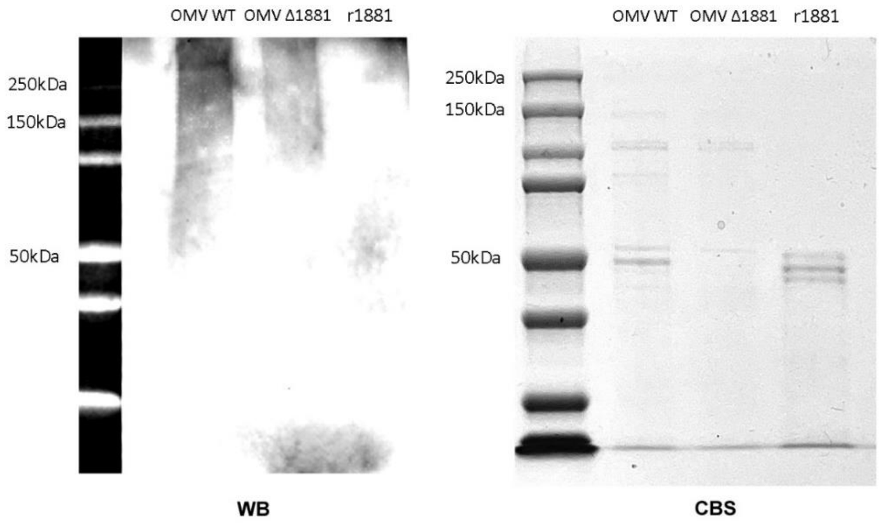

Supplementary Figure S1. Anti-W83 antiserum with the combination of anti-IgM-HRP secondary antibody cannot detect monomeric form of PG1881 (50kDa) in W83 OMVs (OMV WT) and the recombinant PG1881 proteins (r1881) purified from *E. coli,* suggesting that the glycan motif on PG1881 is the IgM binding site. Heat was applied and the band near 50kDa, the monomeric form of PG1881, was absent in the ΔPG1881 mutant OMVs (OMV Δ1881).

Supplementary Figure S2

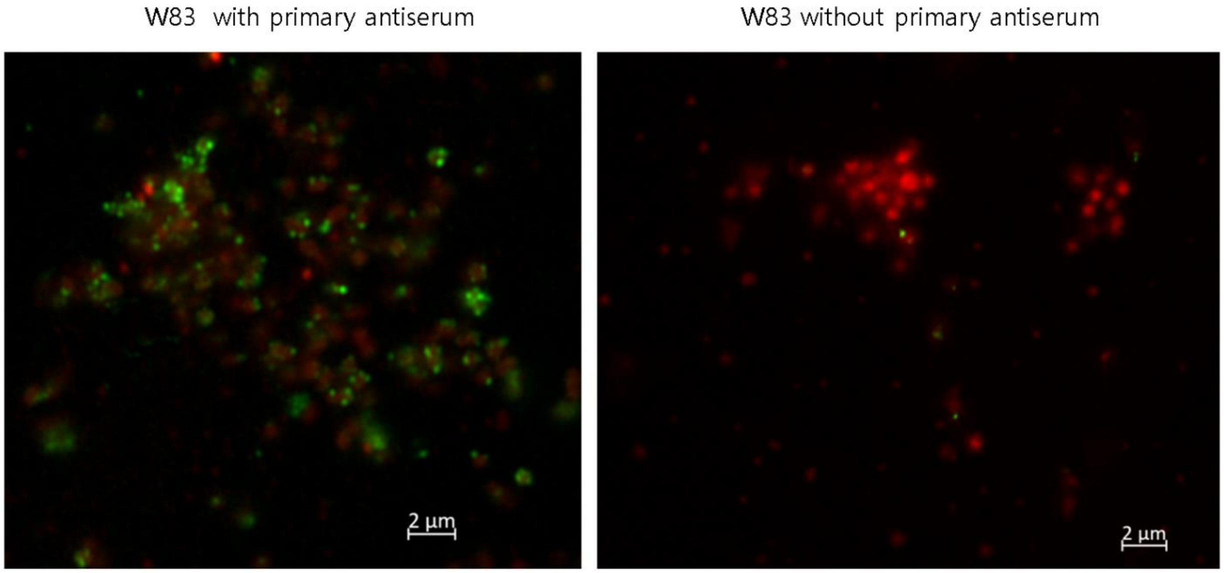

Supplementary Figure S2. No non-specific binding of secondary antibody. Immunofluorescence microscopy images of W83 cells that were treated or untreated with the pre-adsorbed primary antiserum followed by the anti-IgM-AF488 (green). The cells were also stained by the fluorescent nucleic acid stain SYTO85 (red).

Supplementary Figure S3

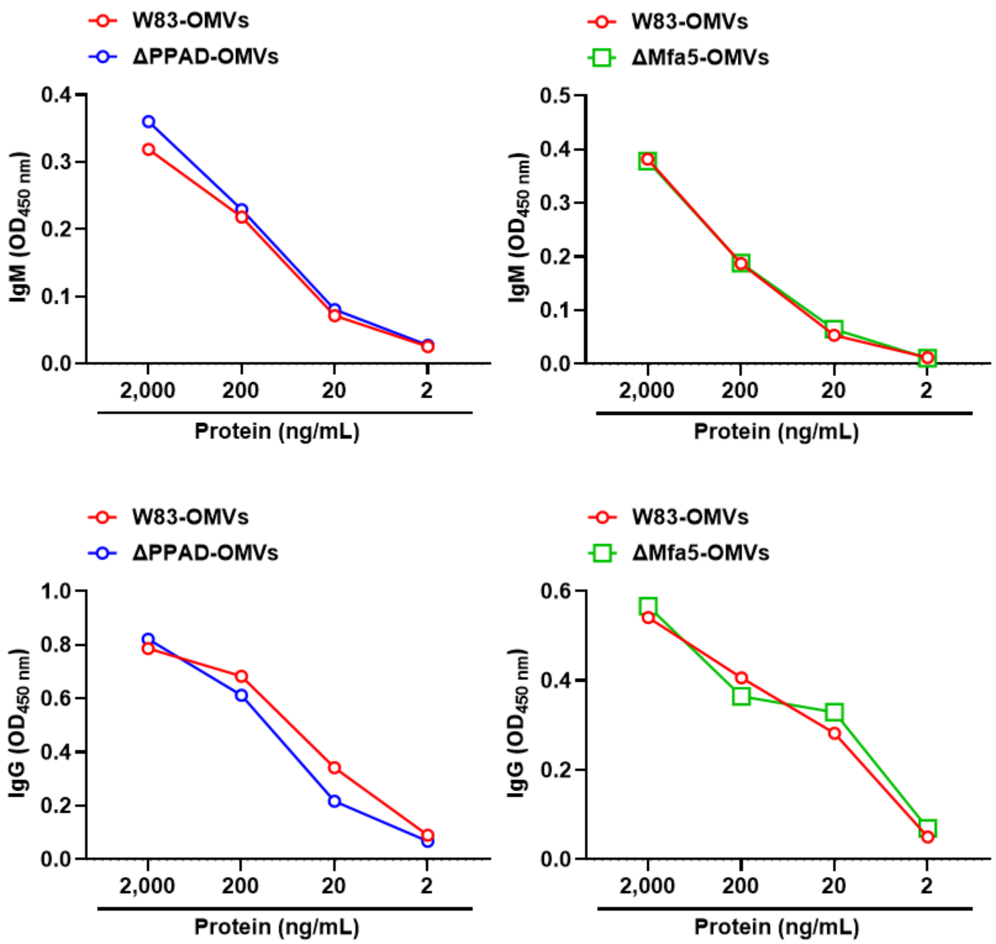

Supplementary Figure S3. Pre-adsorbed IgM shows no major difference in reactivity for crude OMVs isolated from mutants associated with T9SS. ELISA assays using ΔPG1881 mutant cells pre-adsorbed antiserum as the primary antibodies and followed by either IgM-HRP or IgG-HRP as the secondary antibodies to detect the antigens on crude OMVs from WT W83, Δ PPAD and Δ mfa5.

Supplementary Figure S4

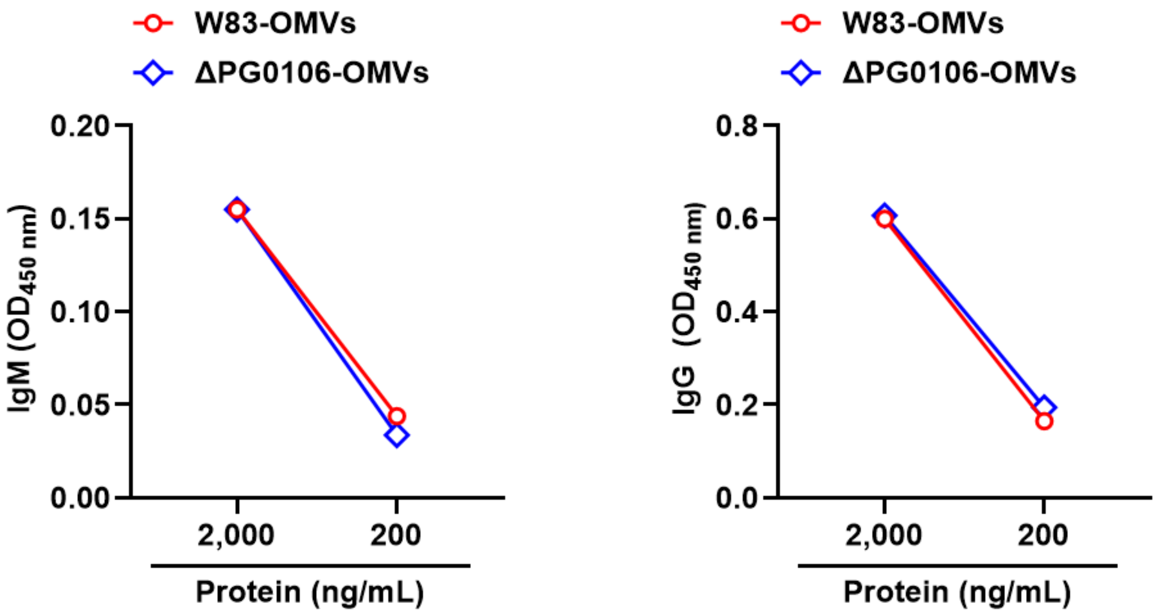

Supplementary Figure S4. Pre-adsorbed IgM shows no major difference in reactivity for crude OMVs isolated from the parent strain W83 and the corresponding K-antigen capsule null mutant ΔPG0106. ELISA assays using pre-adsorbed antiserum as the primary antibodies, followed by either IgM-HRP or IgG-HRP as the secondary antibodies to detect the antigens on crude OMVs from WT W83 and Δ PG0106 mutant.

Supplementary Figure S5

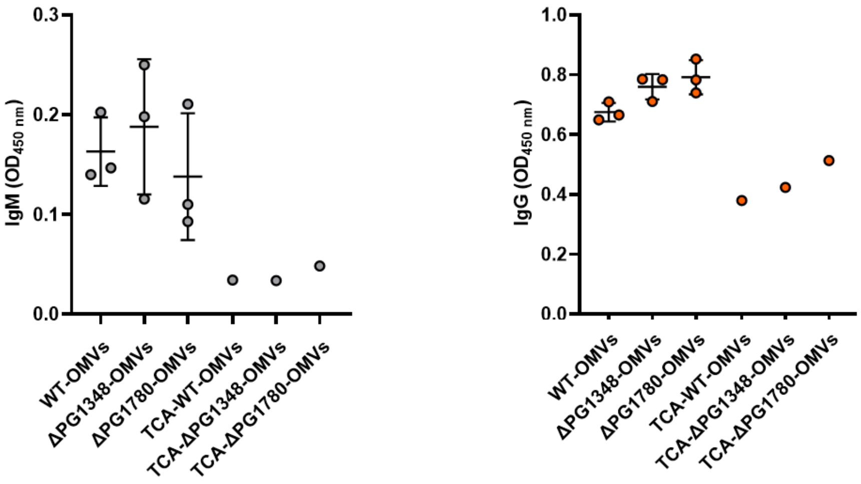

Supplementary Figure S5. Pre-adsorbed IgM shows no major difference in reactivity for crude OMVs and proteins isolated from strains with mutation in genes involved in the synthesis of sphingolipids. ELISA assays using pre-adsorbed antiserum as the primary antibodies, followed by either IgM-HRP or IgG-HRP as the secondary antibodies to detect the antigens on crude OMVs or TCA precipitated proteins from WT W83, ΔPG1348 and ΔPG1780.
